# Controllability affects endocrine response of adolescent male rats to stress as well as impulsivity and behavioral flexibility during adulthood

**DOI:** 10.1038/s41598-019-40061-3

**Published:** 2019-02-28

**Authors:** Maria Sanchís-Ollé, Silvia Fuentes, Jesús Úbeda-Contreras, Jaume F. Lalanza, Arnau Ramos-Prats, Antonio Armario, Roser Nadal

**Affiliations:** 1 Institut de Neurociències, Universitat Autònoma de Barcelona, 08193 Cerdanyola del Vallès, Spain; 2 Animal Physiology Unit (School of Biosciences), Universitat Autònoma de Barcelona, 08193 Cerdanyola del Vallès, Spain; 3 Psychobiology Unit (School of Psychology), Universitat Autònoma de Barcelona, 08193 Cerdanyola del Vallès, Spain; 4 CIBERSAM, Instituto de Salud Carlos III, Universitat Autònoma de Barcelona, 08193 Cerdanyola del Vallès, Spain; 5 Present Address: Laboratory of Sport Psychology, Department of Basic Psychology, School of Psychology, Universitat Autònoma de Barcelona (UAB), Cerdanyola del Vallès, Spain

## Abstract

Exposure to stress during adolescence exerts a long-term impact on behavior and might contribute to the development of several neuropsychiatric disorders. In adults, control over stress has been found to protect from the negative consequences of stress, but the influence of controllability at early ages has not been extensively studied. Here, we evaluated in a rodent model the effects of repeated exposure in adolescent male rats to controllable versus uncontrollable foot-shock stress (CST or UST, respectively). Rats were assigned to three groups: non-stress (stress-naïve), CST (exposed to 8 sessions of a two-way shuttle active avoidance task over a period of 22 days) and UST (receiving the same amount of shocks as CST, regardless of their actual behavior). During adulthood, different cohorts were tested in several tasks evaluating inhibitory control and cognitive flexibility: 5-choice serial reaction time, delay-discounting, gambling test and probabilistic reversal learning. Results showed that the hypothalamic-pituitary-adrenal response to the first shock session was similar in CST and UST animals, but the response to the 8^th^ session was lower in CST animals. In adulthood, the UST animals presented impaired motor (but not cognitive) impulsivity and more perseverative behavior. The behavioral effects of UST were associated with increased number of D2 dopamine receptors in dorsomedial striatum, but not in other striatal regions. In summary, UST exposure during adolescence induced long-term impairments in impulsivity and compulsivity, whereas CST had only minor effects. These data support a critical role of stress uncontrollability on the long-lasting consequences of stress, as a risk factor for mental illnesses.

## Introduction

In humans, exposure to stress during adolescence increases in adulthood the vulnerability to neuropsychiatric disorders, including drug addiction, mood, anxiety, gambling and attention deficit hyperactivity disorders^1–3^. One of the factors that modulates the acute and long-term consequences of stress is controllability^4–6^. Perceived or actual behavioral control over adversity is involved in resilience to stress mediated by individual differences in coping styles, and it may have a profound impact on disease susceptibility^7^. In humans, laboratory uncontrollable stress (UST) (but not controllable: CST) impairs fear extinction^8^ and executive functioning^9^. Moreover, the subjective feelings of helplessness, depression or anger are higher after UST than CST^10,11^. The hypothalamic-pituitary-adrenal (HPA) axis response to stress may also be influenced by controllability, although human data are scarce. Perceived control negatively correlates with cortisol response to a laboratory task^12,13^ and cortisol levels are lower after laboratory CST than UST^11^.

In animal models, the importance of stressor controllability is well recognized since the works of Seligman-Maier and Weiss^14^ using the triadic design including: (i) a “CST” group that can escape from the stressor (originally tail-shock); (ii) a “UST” group that receives the same shock but has no control over it; and (iii) a stress-naive group not receiving shock. The consequences of UST include increased anxiety, decreased social exploration, potentiated fear conditioning and delayed fear extinction^5^. Interestingly, CST does not induce these effects. However, previous studies in rodents indicate that the HPA response is not sensitive to controllability, at least under acute exposure^15,16^. The protective effects of stressor controllability appear to be mediated by a circuit involving the prefrontal cortex (PFC) and the posterior dorsomedial striatum (DMS), which receives important projections from the PFC^5^.

Despite the great attention paid to the influence of control over stress, most studies are focused on adulthood. The acute effects of adolescent CST/UST were studied previously^17^, without important differences in the effects on an escape task. To our knowledge, the long-term effect (adulthood) of stress controllability during adolescence in rodents has only been addressed by Kubala *et al*.^18^ The former study found that a single episode of CST (tail-shock) induces in adulthood an increase in social exploration, in comparison to UST or no stress, suggesting reduced anxiety. These results indicate that adolescent subjects are also sensitive to the long-term effects of controllability. The study of the influence of controllability over stress during adolescence appears to be particularly relevant as this is a period characterized by dramatic changes in PFC circuits that affect both emotional and cognitive functions^19^. Moreover, diminished self-control is a prominent feature of adolescence and during the transition from adolescence to adulthood there is a progressive improvement in executive functioning that parallels PFC maturation^20^. Both human and animal studies indicate that the adolescent brain is markedly sensitive to stress^1,21,22^, especially the PFC^2,23^. Therefore, those PFC-dependent executive abilities may be especially sensitive to the long-term impact of adolescent stress. Previous studies in rodents found that adolescent stress increases adult impulsivity^24^ and decreases behavioral flexibility^25–28^, but the importance of controllability has not been addressed yet.

Several studies indicate that adolescents are more prone than adults to risk-taking/impulsivity behavior^29^. Among other structures, dorsal striatum may be involved in the regulation of behavioral inhibition and risk-taking behavior. Lower availability of dopamine D2 receptors (D2R) in dorsal striatum has been found with positron emission tomography in patients with substance use disorders^30,31^ that negatively correlates with subjective (positive) effects of gambling and impulsivity^32^. In rats, D2R expression in the dorsal striatum showed a U-shaped response relationship with risky decision-making^33^. Dorsal striatum is still under maturation during adolescence in humans^34^. Adolescent rats, but not adults, have a large proportion of neurons in the dorsal striatum activated in anticipation of reward^35^. Within the several subdivisions of the dorsal striatum, the DMS is involved in goal-directed behavior and receives projections preferentially from PFC, whereas the dorsolateral (DLS) is related to habit formation and receives projections preferentially from sensorimotor cortices^36^. DMS may be an area especially sensitive to adolescent adverse experiences as it has been considered important in the development toward the adolescent behavioral phenotype and psychopathology vulnerability^37^. The progressive development of brain circuits involved in impulsivity, behavioral flexibility and risk-taking during adolescence suggests that they may be especially vulnerable to stressor uncontrollability. However, we are not aware of previous studies about the role of controllability/uncontrollability to stress during adolescence and adult vulnerability to impulsivity and compulsivity behaviors, neither in humans nor in rodents.

We then reasoned that repeated exposure to adolescent CST versus UST could permanently shape brain circuits, particularly those related to PFC and dorsal striatum, thereby altering adult behavior. We chose a two-way active avoidance (TWAA) task in which CST rats can control the appearance/duration of foot-shocks, whereas in UST rats’ foot-shocks are independent on their behavior. Avoidance instead of escape was chosen because jumping after receiving foot-shocks is a much more reflexive behavior than jumping during the signal preceding foot-shocks. Our main hypothesis was that exposure to UST (but not to CST) causes long-term impairments in cognitive function in different PFC-dependent tasks: 5-choice serial reaction time (5CSRTT), delay-discounting, gambling and probabilistic reversal learning (PRL). As we observed a more marked impairment in inhibitory control and flexibility in animals exposed to UST, we performed an additional study to measure the short and long-term changes on D2R expression in different subdivisions of the dorsal striatum, including the DMS that is involved in compulsivity/impulsivity-like behavior^38,39^ and is sensitive to stressor controllability^40^.

## Materials and Methods

### Subjects and general procedure

Male Sprague-Dawley rats maintained in standard conditions were used. All protocols were in accordance with the European Communities Council Directive 2010/63/EU and the Spanish legislation (RD53/2013). The experimental protocol was approved by the Ethics Committee at the Universitat Autònoma de Barcelona and the Generalitat de Catalunya. Adolescent stress rats remained undisturbed until tested at adulthood (PND 72) and food-restricted during cognitive tasks. Different cohorts were used for: (i) 5CSRTT, (ii) delay-discounting, (iii) gambling, and (iv) PRL. Two additional cohorts were used to study D2R expression in striatum. Detailed Methods are provided in the Supplementary information.

### Adolescent stress and blood sampling

Rats were randomly assigned to stress-naive (no foot-shocks), CST and UST groups. As our aim was to cover all the adolescent period, the stress (PND 33–55) consisted of 8 sessions (1 daily) given in an unpredictable way throughout a 22-day period. CST rats were exposed to a signaled TWAA session, where they were exposed to a 5 min habituation and then to 50 trials of 10 s of a light-tone conditioned stimulus (CS) immediately followed by foot-shock (unconditioned stimulus, US). Crossing from one side to the other terminated the CS (avoidance) or US (escape) presentation, and was followed by an inter-trial-interval (ITI). UST rats received the same shock, but their behavior had no consequences. Stress-naïve rats were exposed to the shuttle-box without shock. Freezing during habituation was measured in sessions 1, 2 and 8. To study HPA axis activity, blood samples were obtained by tail-nick, 1–3 days before stress (basal levels) and in sessions 1 and 8 (immediately after and 45 min later).

### Biochemical analysis

Plasma ACTH and corticosterone were determined by double-antibody radioimmunoassays that has been extensively validated and used in our laboratory and others^41^.

### 5-choice serial reaction time task

The 5CSRTT evaluates attention, motor impulsivity and perseverative-like behavior^42^. We followed our previous procedure^43^ using 9 rats/group. Briefly, animals were trained during a limited-hold time to nose-poke inside one of five holes after a brief light appeared inside one of them, and then received a pellet. If they failed to respond during this period (omission) or made an incorrect response, the program entered in time-out (TO). The animals were progressively trained across different sessions in which the duration of the hole-light and the hold-time period were progressively decreased^44^. Rats were moved to target conditions of 1 s of stimulus-light duration, 5 s of ITI and 5 s of limited-hold. After two days in those conditions they were trained with ITI = 7 s (17 days), and with ITI = 9 s (8 days) to facilitate the appearance of impulsive responses. The variables measured included: correct, incorrect (errors of “commission”), premature (motor impulsivity), TO (another measure of inhibitory control) and perseverative (additional responses in any hole after a correct response and before collecting the reward) responses and errors of omission. The level of accuracy was: number of correct responses/(number of correct + number of incorrect responses), as a percentage.

### Delay-discounting

This task evaluates cognitive impulsivity as the preference for immediate/smaller over postponed/larger rewards^45^. We used a modification of our previous procedure^43^, with 9–10 rats/group. After pre-training, the proper delay-discounting started. One lever (A, “immediate”) gave 1 pellet after 1 lever press, whereas the other (B, “delayed”) gave 4 pellets. Over days, the reinforcement-delay after pressing B lever was progressively increased (0 s for 10 days, 10 s for 3 days, 20 s for 2 days and 40 s for 2 days). Finally, for 3 additional days the delay was again 0 s to evaluate stability of performance. Each session was divided into five identical blocks of 12 trials, the first six of forced choice, and the last six of “free” choice. The measures in the “free” trials were number of B lever responses and omissions.

### Gambling task

The task assesses decision-making as the rat has to choose between different options differing in the associated reward and penalty (TO)^46^. We used a modification of the procedure of Rivalan *et al*.^46^, with 27–30 animals/group. After pre-training, animals were run in the test. Two consecutive nose-pokes in the same hole were required to get a reinforcer. The contingencies for each hole were: (i) hole A provided 2 pellets (100% trials) with a penalty of 222 s (50% trials); (ii) hole B provided 2 pellets (100% trials) with a penalty of 444 s (25% trials); (iii) hole C provided 1 pellet (100% trials) with a penalty of 12 s (25% trials); (iv) hole D provided 1 pellet (100% trials) with a penalty of 6 s (50% trials). During the TO the hole-light where the response was made remained illuminated and the responses had no consequences. After that, the 4 holes were again illuminated. An identical retest was done one week after. The measures were number of times animals performed two consecutive nose-pokes in a hole to get a reward (“valid” responses) and number of nose-pokes and reinforcers/hole.

### Probabilistic reversal learning

The task evaluates behavioral flexibility in a probabilistic setting, making it more similar to human testing^47^. In this cohort, 8–10 animals/group were used. A modification of the procedure of Dalton *et al*.^48^ was used with the main difference that the final probabilities associated with the “correct” and “incorrect” levers were 95% and 25% respectively. After pre-training, two levers were available at the same time. Once the rat pressed the “correct” lever in 8 consecutive trials, the contingencies were reversed (“reversal”) and the “correct” lever became the “incorrect”. If a rat performed an “incorrect” response or an omission, the rat had to start a new series of 8 “correct” consecutive trials. The TO after an omission lasted 10 s and the ITI lasted 15 s. The experiment finished when the rat performed at least 4 reversals in two consecutive sessions. The measures of cognitive flexibility were the mean number of reversals/session and the number of perseverative errors in the last reversal (number of consecutive incorrect choices after reversal).

### Brain processing and *in situ* hybridization (ISH)

Different sub-sets of animals were perfused 3 days (short-term) or 33 days (long-term) after the end of stress (10 rats/group and period). The protocol for the chromogenic ISH used a D2R riboprobe and was adapted from Simmons *et al*.^49^ Following Paxinos and Watson^50^, the same coordinates were used for each area: Dorsomedial (DMS), Dorsolateral (DLS), Ventromedial (VMS) and Ventrolateral (VLS) striatum. ImageJ public domain image processing software (FIJI v1.47f) was used for quantification.

### Statistical analysis

Data were analyzed with the Statistical Program for Social Sciences, SPSS-IBM (version 24) using a general linear model (GLM) with one between-subjects’ factor (GROUP, three levels: stress-naive, CST and UST). For some variables, within-subject factors were added. To achieve homogeneity of variance, log-transformations were made. If such homogeneity was not achieved, a generalized linear model (GzLM) or a generalized estimating equations model (GEE) was performed. Groups were compared by means of Sequential Bonferroni post-hoc comparisons. All biochemical and ISH samples to be statistically compared were processed in the same assay to avoid inter-assay variability. Alpha level used was p < 0.05 but trends for significance between 0.06–0.05 were also mentioned.

## Results

### Adolescent rats learn the TWAA task across days and freezing behavior did not differ between controllable and uncontrollable stress

The statistical analysis of TWAA indicated that performance was equivalent in the different cohorts. Final sample was n = 54–58/group. Adolescent stress did not modify body weight gain during treatment (stress-naive group: 260.0 ± 7.1 g; CST: 242.4 ± 7.4 g; UST: 244.0 ± 6.7 g; F(2, 164) = 1.87, NS).

**Figure 1:**
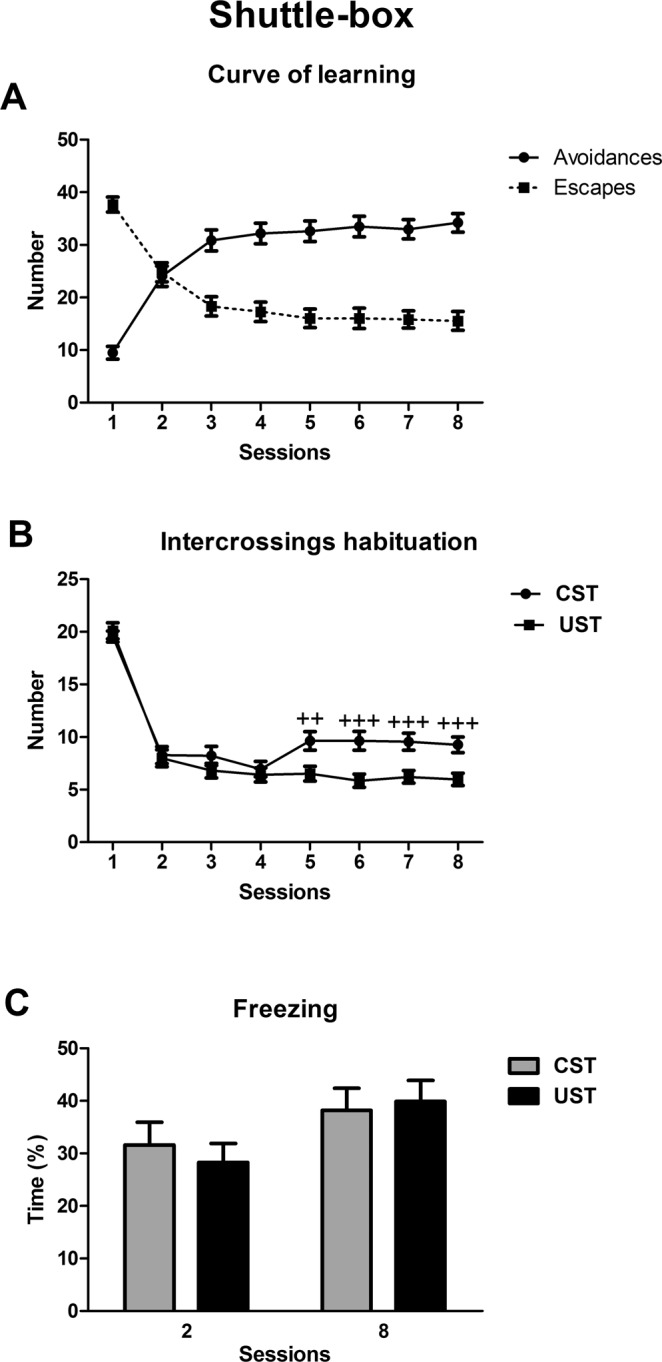
Shuttle-box behavior during the adolescent period. Means and SEM are represented. (**A**) Number of avoidance and escape responses in CST rats (n = 56). (**B**) Number of inter-crossings during the 5 min habituation previous to each shuttle-box session in CST and UST (n = 58) rats. ^++^p < 0.01, ^+++^p < 0.001 versus UST rats. (**C**) Time (%) spent freezing during the habituation period of sessions 2 and 8 in a sub-set CST and UST rats (n = 46–47/group). The effect of time is not represented.

The number of avoidances and escapes during the 8 sessions of stress in CST subjects is shown in Fig. 1A. The GLM analysis showed that the number of avoidances increased, as expected, across days [SESSION: F(7,385) = 79.53, p < 0.001]. The number of avoidances increased by session 5 and kept stable thereafter. The number of inter-crossings during habituation was evaluated as a measure of activity in CST and UST groups (Fig. 1B). The GLM showed a significant effect of SESSION [F(7,770) = 128.1, p < 0.001], GROUP [F(1,110) = 6.46, p < 0.05] and SESSION × GROUP [F(7,770) = 5.51, p < 0.001]. Activity in session 1 (no prior experience with shocks) was similar in the two groups, but differences appeared in sessions 5–8 (between p < 0.01–p < 0.001), with UST rats showing less inter-crossings than CST. More detailed shuttle-box two-way active avoidance behavior in CST rats is provided in the Supplementary information (Suppl. Fig. 1).

As a measure of contextual fear memory, freezing was measured during habituation in sessions 1, 2 and 8. Freezing in stress-naive animals was very low and was not included in the analysis. Freezing was not detected in session 1 as the rats had not experience with shocks, but increased from session 2 to 8 [SESSION: [F(1,91) = 6.32, p < 0.05], without differences between groups (Fig. 1C).

### The endocrine response to adolescent stress was higher after repeated exposure to uncontrollable than controllable stress

**Figure 2:**
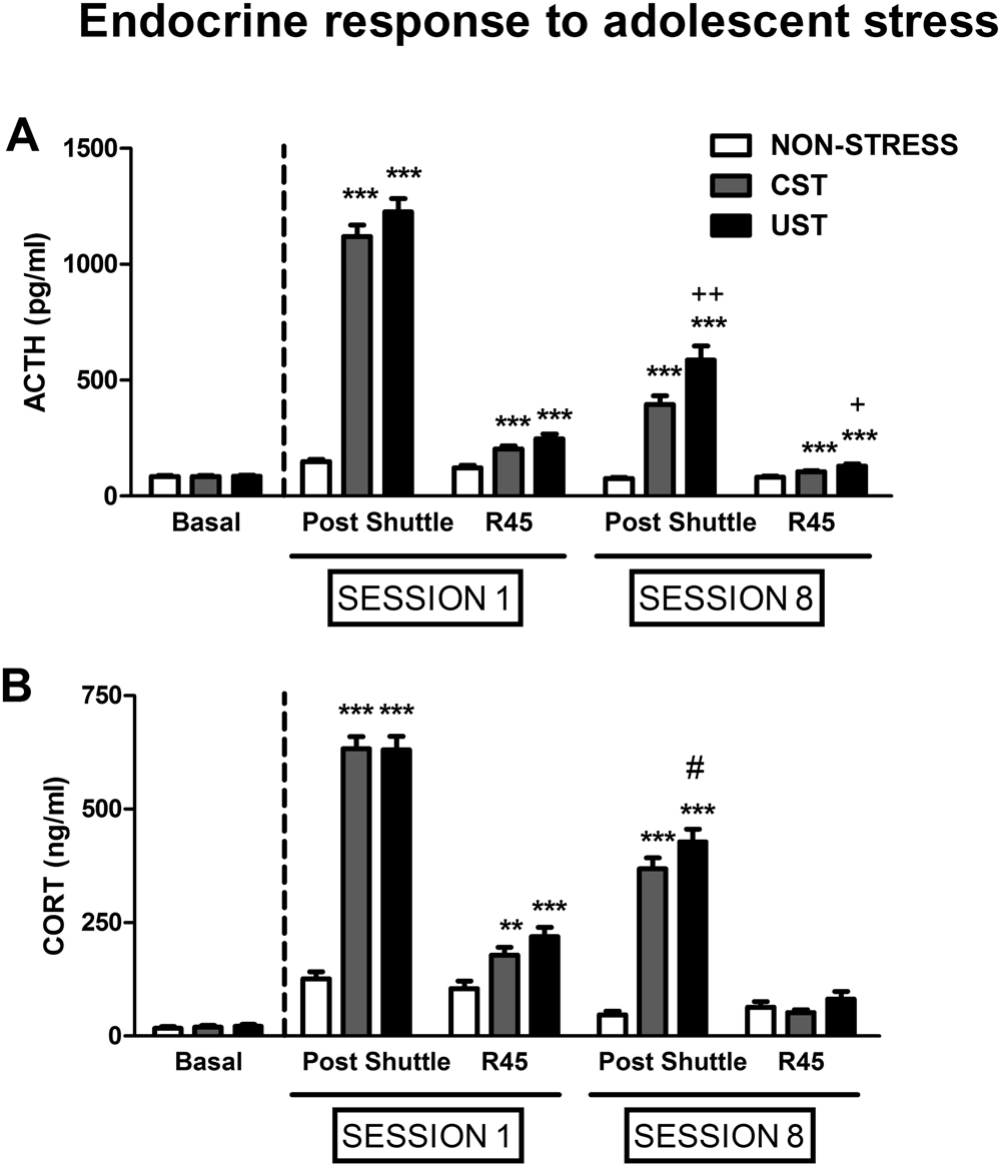
Endocrine response to adolescent stress. Means and SEM of plasma ACTH (**A**) and corticosterone (CORT) (**B**) levels are represented. Rats were assigned to stress-naive, CST and UST groups (n = 29–30/group), and blood sampled under basal conditions (1–3 days before the first session of stress), and after sessions 1 and 8, immediately post-shuttle and 45 min after the end of testing (R45). **p < 0.01, ***p < 0.001: versus stress-naive rats under the same acute condition; ^#^p = 0.053,^+^p < 0.05, ^++^p < 0.01 vs CST rats under the same acute condition. The effect of time is not represented.

As prior statistical analysis revealed that the interaction COHORT × GROUP was not significant, the endocrine response was studied globally. The GEE of plasma ACTH and corticosterone responses to the shuttle included GROUP as between-subjects factor and SESSION and SAMPLING TIME as within-subjects factors. The GEE showed significant effects of all factors and their interactions for both ACTH (Fig. 2A) and corticosterone (Fig. 2B). The GEE analysis of ACTH showed significant effects of GROUP: Χ^2^(2) = 452.26, p < 0.001; SESSION: Χ^2^(1) = 345.45, p < 0.001; SAMPLING TIME: Χ^2^(1) = 708.99, p < 0.001; GROUP × SESSION: Χ^2^(2) = 222.14, p < 0.001; GROUP X SAMPLING TIME: Χ^2^(2) = 654.49, p < 0.001; SESSION × SAMPLING TIME: Χ^2^(1) = 289.11, p < 0.001; GROUP × SESSION × SAMPLING TIME: Χ^2^(2) = 249.08, p < 0.001. The decomposition of the triple interaction indicated that UST rats showed higher ACTH levels than CST rats in the 8^th^ session both immediately after shocks (p < 0.01) and 45 min after (p < 0.05). The GEE analysis of corticosterone showed significant effect of GROUP: Χ^2^(2) = 291.41, p < 0.001; SESSION: Χ^2^(1) = 191.76, p < 0.001; SAMPLING TIME: Χ^2^(1) = 602.27, p < 0.001; GROUP × SESSION: Χ^2^(2) = 51.94, p < 0.001; GROUP X SAMPLING TIME: Χ^2^(2) = 428.39, p < 0.001; SESSION × SAMPLING TIME: Χ^2^(1) = 29.69, p < 0.001; GROUP × SESSION × SAMPLING TIME: Χ^2^(2) = 11.07, p < 0.01. The difference between CST and UST rats in corticosterone levels immediately after the 8^th^ session was marginally significant (p = 0.053). No differences between CST and UST animals were detected during session 1. As expected, both CST and UST rats presented higher ACTH and corticosterone levels than stress-naive at all time periods analyzed (always between p < 0.01–p < 0.001).

Hormonal response to sessions 1 and 8 was correlated with corresponding total shock time. ACTH (but not corticosterone) levels immediately after the 8^th^ but not the first session correlated with shock time in UST rats [r(28) = +0.51, p < 0.01], whereas the correlation did not achieve statistical significance in CST rats [r(27) =+0.36, NS].

### Uncontrollable, but not controllable, adolescent stress increases adult impulsive action in the 5-choice serial reaction time task

**Figure 3:**
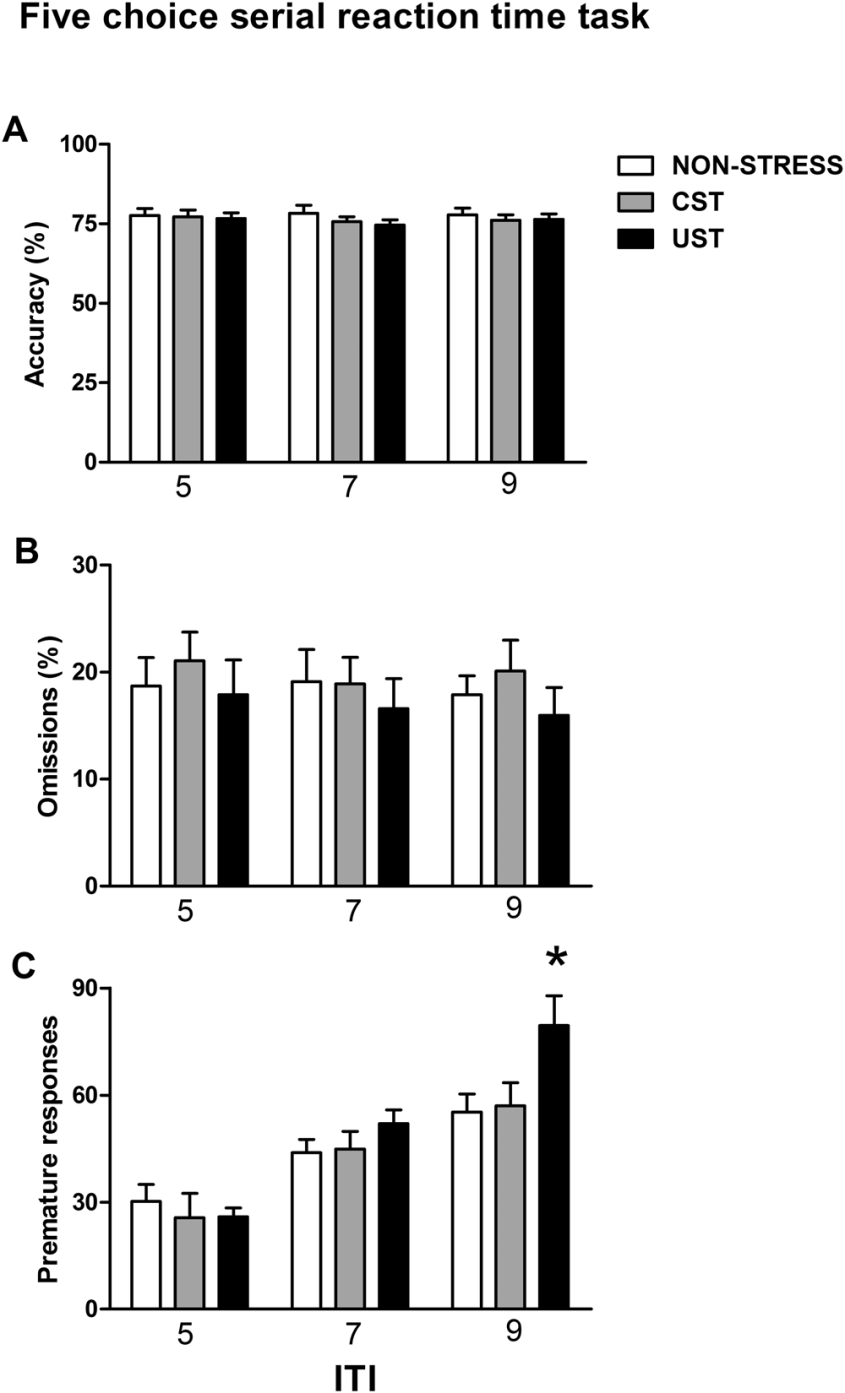
Long-term impact of adolescent stress in the 5-choice serial reaction time task. Rats were assigned to stress-naive, CST and UST groups (n = 9/group) and in adulthood were trained in the five-choice serial reaction time task and exposed to three different inter-trial intervals (ITI) of 5, 7 and 9 s. Means and SEM of the average performance across the different ITIs is represented: (**A**) Accuracy (%), (**B**) Omissions (%) (**C**) Number of premature responses. ^*^p < 0.05 versus stress-naive rats.

The number of sessions needed to achieve appropriate performance did not differ among groups (data not shown). Once in the target conditions (1 s of stimulus-light duration, 5 s of limited hold), accuracy, number of omissions and premature responding with the 5 or 7 s ITI was not different between groups (Fig. 3A–C). In contrast, when the animals were moved to an ITI = 9 s to increase the number of premature responses, differences emerged (Fig. 3C), and a statistically significant GROUP effect was observed [F(2,24) = 4.03, p < 0.05], with the UST animals performing more premature responses than stress-naive (p < 0.05), whereas the CST animals performed similarly to stress-naive. The duration of the session in this stage (stress-naive: 2,114 ± 62 s; CST: 2,193 ± 75 s; UST: 2,553 ± 187 s) was longer in UST than stress-naive rats [GROUP: F(2,24) = 3.72, p < 0.05; comparison between UST and stress-naive: p < 0.05]. No other variable differed among groups.

### Adolescent stress did not affect adult cognitive impulsivity in the delay-discounting test

**Figure 4:**
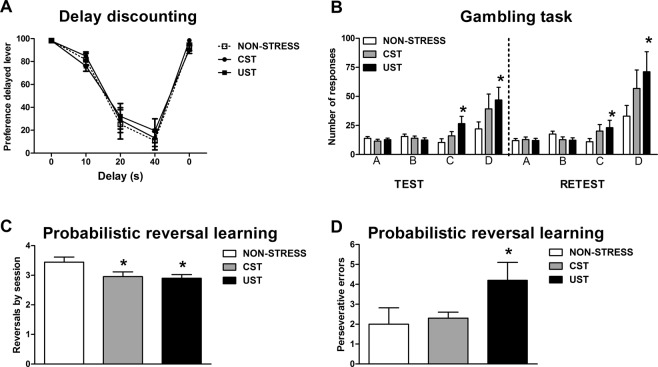
Long-term impact of adolescent stress in different cognitive tasks: delay-discounting, gambling task and probabilistic reversal learning. The rats were assigned to stress-naive, CST and UST groups. In adulthood different cohorts of rats were trained in different cognitive tasks: delay-discounting (n = 9–10/group); gambling task (n = 25–29/group); probabilistic reversal learning (n = 8–10/group). (**A**) Preference (%) for the lever associated to the delayed reward in the delay-discounting task. (**B**) Number of “valid” responses in the gambling task made during a test and a retest in different holes (A,B,C,D) differing in the pellets earned and time-out conditions. ^*^p < 0.05 vs stress-naive rats. (**C**) Number of mean reversals achieved by session in the probabilistic reversal learning task. ^*^p < 0.05 versus stress-naive rats. (**D**) Number of perseverative errors in the last reversal of the last session. ^*^p < 0.05 versus stress-naive rats.

No group differences during pre-training were found (data not shown). The preference for the “delayed” lever decreased with longer delays, but no group differences emerged (Fig. 4A) [DELAY: F(4, 104) = 136.82, p < 0.001; GROUP and GROUP × DELAY: NS]. No differences were found in any other variable.

### Uncontrollable, but not controllable, adolescent stress increases preference for the options with lower time-outs in the gambling task

The number of “valid” responses to each hole (that provided reward) during test and retest is shown in Fig. 4B. The GEE showed significant effects of GROUP [Χ^2^(2) = 8.87, p < 0.05], SESSION [Χ^2^(1) = 10.29, p = 0.001], HOLE [Χ^2^(3) = 20.61, p < 0.001], GROUP × HOLE [Χ^2^(6) = 16.34, p < 0.05] and SESSION × HOLE [Χ^2^(3) = 14.81, p < 0.01], but not GROUP × SESSION and GROUP × SESSION × HOLE. UST rats performed more valid responses in holes C and D (the ones with lower TOs) in comparison to stress-naive, regardless of the session (p < 0.05 in both holes). However, CST rats did not differ from stress-naïve or from UST rats. No other statistically significant differences were found.

### The exposure to uncontrollable adolescent stress has a stronger impact in adult behavioral flexibility in the probabilistic reversal learning

In the mean reversals/session (Fig. 4C) the GROUP factor was significant [F(2,25) = 3.65, p < 0.05] and both CST and UST rats performed less reversals than control ones (p < 0.05 in both cases). Perseverative errors (Fig. 4D) made in the last reversal of the last session showed significant GROUP differences [X^2^(2) = 6.21, p < 0.05], with UST rats making more perseverative errors than controls (p < 0.05), whereas CST animals did not differ from stress-naive. No other statistically significant differences were found.

### Dopamine D2 receptors expression in the dorsomedial striatum was higher after uncontrollable adolescent stress

**Figure 5:**
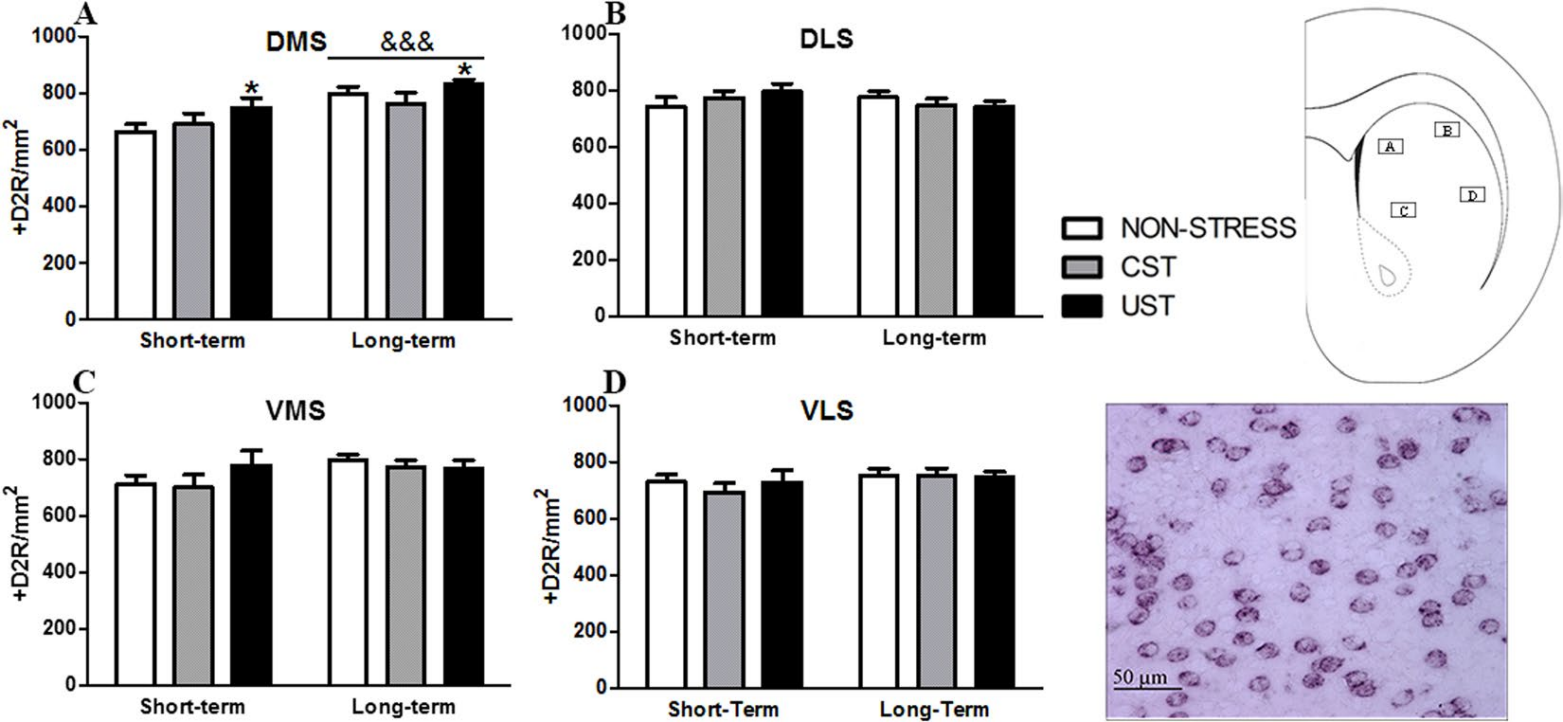
Short-term and long-term impact of adolescent stress on the number of D2R+ cells in the striatum. Rats were assigned to stress-naive, CST and UST groups. Sub-sets of rats were used to study short-term and long-term effects of adolescent stress (n = 9–10/group) (brains perfused 3 and 33 days after the end of stress). Means and SEM of the number of positive dopamine 2 receptors (D2R+) cells/mm^2^ in different subdivisions of the striatum: (**A**) dorsomedial (DMS), (**B**) dorsolateral (DLS), (**C**) ventromedial (VMS) and (**D**) ventrolateral (VLS). *p < 0.05 versus stress-naive rats, independently of age. Schematic localization of the regions analyzed and a representative picture of D2R mRNA staining in the DMS are also provided. Time effect: ^&&&^p < 0.001.

Short-term and long-term effects of stress on the number of D2R+ cells were studied in the different subdivisions of the striatum (Fig. 5). In the DMS, but not in the other subdivisions (DLS, VMS, VLS), the GROUP [X^2^(2) = 6.06, p < 0.05] and AGE [X^2^(2) = 17.05, p < 0.001] factors were significant, the number of D2R+ cells being greater in UST animals than stress-naive (p < 0.05).

## Discussion

In the present study, rats were exposed during adolescence to repeated controllable foot-shock (TWAA) stress (CST group) or to uncontrollable stress (UST), whereas the stress-naive did not receive shock. In the last stress session, UST rats showed higher ACTH levels than CST rats, indicating that repeated experience with controllability is eventually buffering the impact of shocks on the HPA axis. On the contrary, the development of contextual fear conditioning was not affected by controllability. In adulthood, animals exposed to UST displayed increased impulsive action, a preference for options with shorter TO and several signs of behavioral inflexibility. Those responses were accompanied by an increase in D2R expression in DMS. The impact of UST was not due to a generalized impairment, since other cognitive measures were not affected. The long-term effects of exposure to CST were very mild and restricted to a decrease in reversals without impact in the number of perseverative errors. Globally, our data support the importance of the psychological component of stressors (and not only their physical properties) and indicate that controllability mitigates the long-lasting negative impact of stressors in adolescent rats.

### Response of adolescent rats to controllable and uncontrollable stress

Repeated exposure to CST/UST during adolescence neither reduced body weight gain (present data) nor altered relative adrenal weight (unpublished), parameters altered by chronic severe stressors^51^. This suggests that the procedures were not particularly severe. The response of HPA hormones was studied during the first and last sessions. A clearly reduced HPA response was found in both CST and UST animals in the last session compared with the first one, likely reflecting the lower number/duration of shocks because of learning to avoid. More importantly, no differences between CST and UST rats were observed in ACTH and corticosterone responses to the first session, whereas in the last session ACTH levels were lower in CST than UST rats and the same trend was found for corticosterone. Therefore, the influence of control was not evident during the first session but appeared when animals had repeated experience with the procedure. The existence of HPA differences in the present study between CST and UST rats with several days of experience with the task and not in the first session gives support to the proposal that the “experience of controllability” develops across time^52^. Interestingly, the correlation between ACTH and total time of shock during session 8 was significant in UST but not in CST rats. As ACTH response appears to be a good marker of stressor intensity^53^, the lack of a significant correlation in CST rats suggests that controllability adds complexity to the expected relationship between the intensity of stressors and HPA response.

These results are consistent with rodent literature. Using procedures in which shock was not preceded by any signal and only escape from shocks was possible, HPA response did not differ between CST and UST groups. This has been observed after a single session of tail-shocks in rats^14,15^ or after repeated foot-shock in mice and rats^54,55^. In contrast, using procedures in which a signal preceded foot-shocks, thereby allowing the animals to impede the appearance of the aversive stimulus (avoidance), UST rats showed higher resting corticosterone on the day after the 6^th^ session than CST rats that not differed from stress-naive rats^56^, suggesting a lower impact of CST after repeated experience of control. Human data about HPA response and objective or perceived control over stress^11–13^ have been obtained after acute conditions and future studies are needed in other conditions.

The freezing response to the shuttle-box during habituation, a measure of contextual fear memory, was assessed during sessions 2 and 8. As expected, freezing was undetectable in stress-naive rats, whereas both CST and UST rats developed freezing, which was similar in both conditions. However, between sessions 5 and 8, inter-crossings during habituation were greater in CST than UST rats. Whether this reflects lower levels of fear to the context in CST rats (this parameter being more sensitive to minor differences in fear than freezing) or a reinforced response to move to the other compartment even in the absence of signals announcing shocks is unclear. The finding that intercrossings during the habituation was essentially similar between sessions 2 and 8 whereas intercrossings during the ITIs progressively increased favors the hypothesis of lower levels of fear in CST rats, in accordance with prior reports in adult rats using a CST-UST design also involving a TWAA task in which fear conditioning to the context was evaluated several days after the procedure^52,56^. The development of fear conditioning after CST/UST has also been evaluated by Liu *et al*.^57^ and Pryce *et al*.^58^, who studied in adult mice freezing across different sessions of escape from foot-shocks, obtaining surprisingly that CST subjects presented similar or more contextual fear memory than UST subjects. Taken together, results strongly suggest that differences in the controllability paradigm (escape versus avoidance/escape) may be critical.

### Long-lasting effects of uncontrollable adolescent stress

Exposure to uncontrollable (but not controllable) stress during adolescence induced a long-lasting impairment of inhibitory control as evidenced by an increase in premature responding in the 5CSRTT when the task implied longer ITIs. These data agree with previous studies using chronic unpredictable stress during adolescence^24^. This increase in impulsive action was dissociated from impulsive choice, as performance in delay-discounting was not affected, in accordance with previous reports using another model of stress^59^. Similarly, social isolation during adolescence also increases premature responding in the 5CSRTT without affecting delay-discounting in adulthood^60^. The dissociation between both types of impulsivity has been previously described as indicative of the multidimensional nature of impulsivity^45^. The increase in premature responding observed in UST rats was accompanied by a preference for the options associated with shorter TO (C and D) in the gambling task, supporting an impairment of inhibitory control. Unexpectedly, stress-naive rats in our current conditions did not prefer overtly the “advantageous” options (C and D), which opens the possibility that with other versions of the task^61^ or other experimental conditions the long-term impact of UST will be different. To our knowledge, no previous reports have addressed the impact of adolescent stress in gambling tasks during adulthood, and more research is needed in this area.

The impairment of inhibitory control in UST rats was accompanied by perseverative-like behavior. In the PRL, the UST rats performed less reversals and more perseverative errors. This impairment in behavioral flexibility is in line with results obtained after foot-shock^25^, adolescent social defeat^26,27^, and chronic unpredictable stress^28^, although the impact may be dependent on genotype^25^ and the specific period of exposure during adolescence^27^.

In contrast to the effects of UST, the impact of CST was only restricted to some impairment in the PRL, without effects in other tasks. These data agree with Lucas *et al*.^56^, who using a repeated exposure to a similar TWAA in adult animals observed that controllability ameliorated the impact of UST in the forced swim test and olfactory discrimination. Globally, these results support that controllability may buffer the negative impact of UST^5^ and extend previous data in adult animals with models of acute stressors to adolescent repeated stressors.

The impairment of executive functioning observed in UST rats (impulsive/compulsive-like behavior) was accompanied by an increase in the number of D2R+ neurons in the DMS, but not in other striatal subdivisions. The effect was observed shortly after stress and maintained in the long-term. To our knowledge, this is the first work addressing the specific contribution of CST/UST to changes in D2R+ neuronal populations in the dorsal striatum. Striatal projection neurons (medium-sized spiny neurons or MSNs) are subdivided in two types in function of their axonal projections and neurochemical expression patterns^62^. Those MSNs expressing D1R form the direct pathway that project to the *substantia nigra pars reticulata* and internal *globus pallidus*, whereas those expressing D2R form the indirect pathway that project indirectly to the *substantia nigra* by way of the external *globus pallidus* and the subthalamic nucleus. D1R are positively coupled to adenylate cyclase and causes electrophysiological activation of neurons thus activating the direct pathway, whereas D2R exerts opposite effects thus inhibiting the indirect pathway.

The results with the DMS are particularly interesting because this nucleus takes part in a circuit essential for inhibitory control^38^. Within the DMS, local injection of D2R agonists increases premature and perseverative responding in the 5CSRTT, without affecting accuracy^63^. In contrast, the activation of D1R increased premature responses only at doses that impaired accuracy/omissions, without affecting perseverative responses^63,64^. The effects of both, D1R and D2R ligands, could be task-dependent and play different roles in the stop-signal reaction time procedure^65^. The increase in premature/perseverative responses after D2R activation^63^ is consistent with the present observation of increased expression of D2R in UST rats and also with electrophysiological data indicating that putative MSNs of the indirect pathway tend to fire more strongly during “no-go” trials^66^. Enhanced inhibition of D2R+ neurons might reduce their capability to engage the “no-go” process thus increasing impulsivity and perhaps compulsivity. In any case, the role of dopamine and its receptors appears to be complex and can be dependent on the specific area of the brain targeted and the characteristics of the tasks^39,67.^

As mentioned, in the present study the exposure to adolescent UST increased D2R+ in DMS but not in DLS, at the same time that increased motor impulsivity but not cognitive impulsivity. Previous data indicate that DMS lesions increase impulsive and compulsive-like behavior in the 5CSRTT whereas DLS lesions were ineffective^68^. In contrast DLS lesions increase cognitive impulsivity in a delay-discounting procedure^69^, task not affected in our present data. To our knowledge there are no previous studies about the effects of DMS lesions in cognitive impulsivity tasks.

Maier’s laboratory has demonstrated that the protective effects of behavioral control towards a stressor seem to require DMS but not DLS functioning^40^. Our results are also compatible with the hypothesis that in adults repeated stress favors habitual over goal-directed actions^70^, at least in part by reducing dendritic complexity of PFC and DMS neurons and increasing dendritic arborization of DLS neurons^71^. Globally present data suggest that the MSNs of the indirect pathway (expressing D2 receptors) arising from DMS are specifically affected, in a long-lasting way, by exposure to uncontrollable (but not controllable) stress during the adolescent period. Results support also that DMS is of particular importance in the disorders that involve a transition from goal-directed actions to habitual responding, such as drug addiction, pathological gambling and other impulsivity/compulsivity related disorders.

Although the present data support an influence of adolescent stress on PFC-dependent tasks, the impact of UST is mild and restricted to specific measures of executive functioning, highlighting the possibility that the plasticity of the adolescent brain may partially overcome the possible negative impact of stress. Future studies are needed to address whether adult exposure to the same stressors regime would induce equivalent long-term impact in the studied parameters. A recent work^72^ has directly compared long-term effects of adolescent and adult sensitivity to chronic stress, but the results do not allow to achieve any general conclusion on a putative differential vulnerability because the various dependent variables did not follow the same pattern. To definitively study whether the effects of the observed study are independent or not of adolescence we need additional works comparing in the same conditions the impact of adult versus adolescent stress.

A limitation of the present study is that only male rats were studied, and recent data indicate that the protective effects of controllability are not detected in females^73,74^. Another question that needs to be addressed is whether only one single stress exposure would exert the same effect as our paradigm (eight exposures across the entire adolescent period), as it has been studied that a single session of tail-shocks during this stage may induce long-term effects^18^. Finally, only D2R in dorsal striatum were analyzed and not in the PFC, as in our present conditions the expression of D2R in the PFC was very low and could not be evaluated, and other markers in addition to D2R should be also explored.

In conclusion, our results support human data indicating that adolescent (uncontrollable) stress is a risk factor for neuropsychiatric disorders in adulthood^1–3^. Interestingly, although the HPA axis response did not differ initially between UST/CST, after repeated exposure a lower response was observed in those animals having control over stress. A stronger impact of UST (and not CST) was observed in the long-term in impulsivity/compulsivity-related measures, thus suggesting a protective long-lasting effect of control over stress. Although the neural circuits and neurochemical processes underlying this differential effect remain to be studied, the ability to control threats in our environment appears to be critical for behavioral resilience, and this is particularly relevant during adolescence, a critical developmental window of heightened sensitivity to stressors. Preventive approaches in humans should aim at increasing perception of control as a way to decrease vulnerability to some psychopathologies with impulsivity/compulsivity-related symptoms.

## Supplementary information

Supplementary information
