# Supplementary material for: Controllability affects endocrine response of adolescent male rats to stress as well as impulsivity and behavioral flexibility during adulthood
Source: Sci Rep. 2019 Feb 28;9:3180. doi: 10.1038/s41598-019-40061-3 (PMC6395608; doi:10.1038/s41598-019-40061-3)
Supplement: Supplementary file 1 — Supplementary information [file 41598_2019_40061_MOESM1_ESM.docx]

**Controllability affects endocrine response of adolescent male rats to stress as well as impulsivity and behavioral flexibility during adulthood**

**SUPPLEMENTARY METHODS AND RESULTS**

**Maria Sanchís-Ollé^1, 2^; Silvia Fuentes^1,3^; Jesús Úbeda-Contreras^1, 2^, Jaume F Lalanza^1^; Arnau Ramos^1,2^; Antonio Armario^1,2,4^ * and Roser Nadal ^1,3,4^ ***

1. **Institut de Neurociències**
2. **Animal Physiology Unit (School of Biosciences)**
3. **Psychobiology Unit (School of Psychology)**
4. **CIBERSAM, Instituto de Salud Carlos III**

**Universitat Autònoma de Barcelona; 08193 Cerdanyola del Vallès; Spain**

*** Both authors contributed equally**

**To whom the correspondence needs to be addressed:** [**Roser.nadal@uab.es**](mailto:Roser.nadal@uab.es)

Present address:

**JF, Lalanza**: Laboratory of Sport Psychology

Department of Basic Psychology

School of Psychology

Universitat Autònoma de Barcelona (UAB), SPAIN

**Subjects**

Male Sprague-Dawley rats [Crl:OFA(SD)] bred at the Universitat Autònoma de Barcelona Animal Facility were used. Animals were bred in house in a side by side building and transported to the Behavioural Facility in a less than one-minute transport after weaning (PND 21). Only 1-2 pups from the same mother were included in each group. Rats were housed in pairs in Makrolon transparent polycarbonate wire-topped cages with a solid bottom (26.5 x 42.5 x 18.5 cm, Ref. 1291 Eurostandard Type III H) containing sawdust bedding (Lignocel 3/4, Harlan) in a climate-controlled environment at 20–21 °C on a 12-h light–dark cycle (lights on 8:00 am) and fed with a regular diet (Ref. 2014C Teklad Global 14% protein, Harlan). Animals were allowed ad libitum access to food except when mentioned in adulthood. Rats always had free access to filtered tap water. No specific environmental enrichment program was used in the animal facility. All animal protocols were in accordance with the European Communities Council Directive 2010/63/EU and the Spanish legislation (RD53/2013) and were approved by the Ethics Committee for Human and Animal Research of the Universitat Autònoma de Barcelona, and by the Catalan Government (Generalitat de Catalunya). A maximal effort was expended to minimize the suffering of the animals and the number of animals used. The animal’s health was monitored daily. Subjects were exposed to handling for 3-4 days before the beginning of the adolescent stress and for 2-3 days before the beginning of the adult cognitive testing.

**General procedure**

Animals were exposed to stress during adolescence. The treatment started at approx. postnatal day (PND) 33 and finished at PND 55. Rats were exposed to controllable (CST group), uncontrollable (UST group) or no stress (stress-naive, control group). The stress consisted of 8 sessions (1 daily) given in an unpredictable way (time of the day and interval between sessions was changed at random) throughout a period of approx. 22 days. In sessions 1 and 8, some animals were blood-sampled to study the hypothalamic-pituitary-adrenal (HPA) response to the test (see below). Both animals of the same home-cage received always the same treatment.

After adolescent stress animals remained undisturbed until tested. Adult testing started at approx. PND 72. Different cohorts were used to evaluate these tasks: (i) 5-choice serial reaction time (5CSRTT), (ii) delay-discounting, (iii) gambling, and (iv) probabilistic reversal learning (PRL). These tasks were selected because executive abilities, depending on prefrontal cortex functioning^1^, may be especially sensitive to the long-term impact of adolescent stress. Two additional cohorts were used to study dopamine receptors type 2 (D2R) expression in the dorsal striatum. In the adult cognitive tasks animals were food-restricted to the 60-70% of their daily food intake, allowing an increase in body-weight across weeks. Rats were run for 5-7 days/week. All operant chambers were carefully cleaned between rats with a soap-containing solution.

**Apparatus**

*Shuttle-box*

The adolescent stress was administered in 4 different shuttle-boxes. Each shuttle-box (Ref LE916, Panlab-Harvard, Barcelona, Spain) was divided into two equally sized compartments (25 × 25 × 25 cm), connected by an opening door of black PVC (8 cm wide and 10 cm high). The frontal wall of each compartment acted as a door and was made of clear Plexiglas, the other 3 walls were of black pvc and the roof was of aluminium and Plexiglas. The chambers had a waste pan and the floor in each compartment was composed of 19 stainless steel rods (3 mm in diameter), spaced 1 cm from center to center, and wired to a shock generator with scrambler (Ref LE10026). Each compartment had a light (4 cm diameter 2.4-W, 24-V) centered in the back wall at 19.5 cm from the floor and the box was provided with a general sound generator. Each shuttle-box was placed inside a metallic sound-attenuating box (Ref LE26, 66 x 50 x 55 cm) provided with a dim light and a fan that helped to mitigate strange sounds and provided a background noise of 60 dB. The chambers were connected to an interface (Ref LE900) and controlled by the Packwin software version 1.2 (Panlab-Harvard, Barcelona, Spain). Behavior was also monitored by a miniature camera (Ref. KPC-S500P3, KT&C co.) mounted on the front panel of each compartment.

*5-choice serial reaction time boxes*

The 5CSRTT was performed in 2 operant boxes provided with nose-pokes (Ref MED-NP5M-D1, Med-Associates Inc., St. Albans VT, USA). The chambers (external dimensions: 31.8 x 25.4 x 34.3 cm, “extra-tall”) had two walls made of aluminium and two walls made of clear Plexiglas. One of the walls was curved and contained 5 holes (2.5 x 2.5 cm) that were 2.4 cm deep and 2.4 cm apart, placed 6 cm from the floor. Each hole had an infrared (IR) detector (1 cm from aperture) for nose-poke and a light inside (LED, 6.4 mm in diameter). The chambers had a stainless steel grid floor (0.5 cm diameter) and a waste pan. The pellet dispenser was connected to a food magazine (5.4 x 5 cm) with a light inside (1 cm in diameter) and a head entry detector (IR) that was placed in the opposite wall from the holes. Each hole was placed 33 cm from the food magazine. The house-light (28 V DC, 100 mA) was placed 30 cm from the floor, near the roof and above the food magazine. The chambers were placed inside a PVC sound-attenuating cubicle (63.5 x 40 x 60.5 cm) with a fan that provided a background noise of 60 dB. The chambers were controlled by the Med-PC software (version IV). Behavior was also monitored by a miniature camera (Ref. KPC-S500P3, KT&C co.) mounted on the front panel.

*Delay-discounting and Probabilistic reversal learning boxes*

These tasks were performed in 8 operant boxes (Ref LE1005, Panlab-Harvard, Barcelona, Spain). Each chamber (25 x 25 x 25 cm) had a clear Plexiglas door and black PVC sidewalls. The floor was composed of 19 stainless steel rods (3 mm in diameter), spaced 1 cm from center to center. A house-light (4-cm diameter 2.4-W, 24-V) was placed in the right wall at 22 cm from the floor. In the left wall, two metal retractable response levers (6 cm above the floor) were placed on either side of a food magazine (3.5 x 3.5 cm). Another light was placed above each lever and above the food magazine (4 lights in total). The chambers were placed inside a metallic sound-attenuating box (Ref LE26, 66 x 50 x 55 cm) provided with a fan that helped to mitigate strange sounds and provided a background noise of 60 dB. The software (Packwin 2.00.2, Panlab-Harvard) controlled the administration of the different stimuli and recorded the data. Behavior was also monitored by a miniature camera (Ref. KPC-S500P3, KT&C co.) mounted on the front panel of the sound-attenuating box.

*Gambling boxes*

The gambling task was performed in other different 4 operant boxes (Ref LE1005, Panlab-Harvard, Barcelona, Spain) provided with nose-pokes. The lateral walls were made of black PVC, the frontal and back walls of clear Plexiglas and the roof of aluminum and Plexiglas. In the left wall were placed the food magazine provided with an IR detector and a house-light 4 cm above the magazine. In the right curved wall were placed 5 holes to be illuminated (LED) (2.4 cm diameter, 1.8 cm deep, separated each one 2.5 cm, the center hole was blocked) provided with IR detectors at 2.8 cm from the floor. The rest of the characteristics of the boxes were similar to the delay-discounting/PRL ones, except that no levers were available.

**Procedure**

*Adolescent stress and blood sampling*

The CST rats were submitted to a signaled (auditory/visual) TWAA^2^ that combines Pavlovian and Instrumental learning^3^. The paradigm allows to characterize proactive and reactive coping strategies to stressful situations and it has been recently used in translational studies in human populations as a tool to evaluate individual vulnerability to stress^4^.

The test was performed in a black room different from the laboratories used for cognitive testing and for housing. Rats were exposed to a 5 min period of habituation without stimuli and then to 50 trials of 10 s of conditioned stimulus (CS, light of 7 W and sound of 2400 Hz at 80 dB, presented simultaneously), immediately followed by a scrambled electric shock (unconditioned stimulus, US, 0.7 mA, 10 s, non-continue squared current, frequency: 20 Hz, each pulse duration 8.3 ms, polarity: monophase, pulse current: effective) administered through the metal grid floor of the box. Crossing from one side to the other compartment terminated the CS (i.e. avoidance response) or US presentation (escape response), and was followed by a variable interval (VI) 30 s (range 5-55 s) pause (inter-trial interval, ITI). Seven more sessions were administered with an identical procedure. Main outcome variables were: avoidance, escape and null responses, habituation and ITI crossings, and avoidance, escape and response latencies. UST rats received the same amount of shock than CST rats but their behavior had no programmed consequences (i.e. they were not able to avoid or escape from the shock). Stress-naive rats were only exposed to the shuttle-box with no shock.

Freezing behavior during the 5 min of habituation to the shuttle-box in sessions 1, 2 and 8 was measured from the video-recordings only in some of the cohorts (5CSRTT, delay-discounting and gambling). The purpose of this measure was to study the possible development of differential contextual fear memory between CST and UST groups. Freezing involved the absence of all movements other than respiratory-related movements^5^.

To study the activity of the HPA axis (adrenocorticotropic hormone or ACTH and corticosterone), blood samples were extracted by tail nick. The tail nick consisted of gently wrapping the animals with a cloth, making a 2 mm incision at the end of the tail veins and then massaging the tail while collecting, within 2 min, 300 μl of blood into ice-cold EDTA capillary tubes (Stardest, Granollers, Spain). The cage-mates were sampled simultaneously (two experimenters were sampling at the same time and a third was gently holding the two rats). This procedure is extensively used in our lab and by others because low resting levels of hormones are obtained^6^. Basal levels were taken 1-3 days before the first session of stress. Blood sampling was also performed in shuttle sessions 1 and 8 at different times: immediately at the end of stress (approximately 40 min after the introduction into the shuttle-box) and 45 min after. Stress-naive animals were blood sampled at the same time-points. Only some animals were sampled: cohorts 5CSRTT, delay-discounting and a sub-set of animals from the gambling cohort. The final sample from the endocrine analysis was: n=29 for the stress-naive group; n=29 for the CST group and n=30 for the UST group.

*Biochemical analysis*

Plasma ACTH and corticosterone levels were determined by double-antibody radioimmunoassay (RIA), as previously described^7^. Briefly, ACTH RIA used 125I-ACTH (PerkinElmer Life Science, Boston, USA) as the tracer, rat synthetic ACTH 1–39 (Sigma, Barcelona, Spain) as the standard and an antibody raised against rat ACTH (rb7) kindly provided by Dr. W.C. Engeland (Department of Surgery, University of Minnesota, Minneapolis, USA) and a non-equilibrium procedure was followed. Corticosterone RIA used 125I-corticosterone-carboximethyloxime-tyrosine-methyl ester (ICN-Biolink 2000, Barcelona, Spain), synthetic corticosterone (Sigma, Barcelona, Spain) as the standard and an antibody raised in rabbits against corticosterone–carboximethyloxime-BSA kindly provided by Dr. G. Makara (Institute of Experimental Medicine, Budapest, Hungary) and an equilibrium procedure was followed. The intra-assay coefficient of variation was less than 6% for ACTH and corticosterone. The sensitivity was 12.5 pg/ml for ACTH and 1 ng/ml for corticosterone.

*5-choice serial reaction time task*

The 5CSRTT was used to evaluate attention, impulsive action and perseverative-like behavior^8^. In this cohort, 9 stress-naive, 9 CST and 9 UST animals were used. We followed a procedure similar to the one used in our previous study^9^. The session took place in a darkened room. Pellets from Bio-Serv (45 mg, Ref. F0021) were used as a reinforcer. Each session started with the illumination of the house-light and the magazine-light and the administration of a pellet. When the rat collected the pellet inside the food magazine, the magazine-light was extinguished and an ITI was started. Responding to the food magazine during the ITI restarted it. After the ITI, a brief light inside one of the five holes was provided. If the animal performed a correct response during a limited-hold time, the animal received a pellet, and the magazine-light was again illuminated. When the animal made a correct response, the stimulus-light was extinguished. If the animals failed to respond during this limited-hold period (omission) or made an incorrect response, the program entered in a time-out (TO) of 5 s during which the rat did not receive a reward and the house-light was OFF. Responses in any hole before the light appearance also produced a TO. The responses in the holes made during the TO also re-started the TO. Responding to the food magazine during the TO initiated a trial. After the TO, the house-light and the magazine-light were again illuminated, and the rat had to nose-poke inside the food magazine to start a new trial. The stimulus-light was presented the same number of times in each hole during the complete session in a random order. At the end of each session, all lights were switched OFF.

The animals were progressively trained across different sessions in which the limited-hold period and the duration of the hole-light were progressively decreased^10^. Rats were trained to criteria until they arrived at the following target parameters: 1.25 s of stimulus-light duration, 5 s of ITI and 5 s of limited-hold. Later, all rats were moved to target conditions of 1 s of stimulus-light duration, 5 s of ITI and 5 s of limited-hold. After two days in those conditions they were trained with 7 s of ITI for 17 days, and with 9 s of ITI for 8 days to facilitate the appearance of impulsive responses during the ITI. Until the stage of 9 s of ITI, each daily session consisted of 90-100 trials or 30 min of testing, whichever was first achieved. In the stage of 9 s of ITI the maximum duration of testing was increased to 45 min.

The variables measured included: correct responses, incorrect responses (errors of "commission"), premature responses (anticipatory responses made during the ITI, as a measure of motor impulsivity), responses during the TO (as another measure of inhibitory control), perseverative responses (as a measure of compulsivity: an additional response performed in any hole after a correct response before collecting the reward, these responses did not produce TO) and errors of omission (when no response was made during the limited-hold period, measure that reflects motivational/motor deficits). The level of accuracy (as a measure of sustained and spatially divided visual attention) was calculated by the following formula: number of correct responses/(number of correct + number of incorrect responses) expressed as a percentage. The other parameters measured were: number of trials finished, duration of the session, latency of the correct and incorrect responses and latency to collect the reward. All the testing lasted as a mean 143 days (including weekends with no testing).

*Delay-discounting*

The delay discounting task evaluates cognitive impulsivity as the tendency to choose immediate smaller rewards over larger but postponed ones^11^. In this cohort, 10 stress-naive, 10 CST and 10 UST animals were used. One subject from the stress-naive group was removed because it was not able to learn the task. We used a modification of our previous procedure^9^. The reward were 45 mg pellets (Ref F0021, Bioserv). The procedure involved 3 phases of pre-training. In the first phase, overnight sessions were conducted with the house-light always ON and the two levers accessible (not retracted) and active to provide 1 pellet after 1 lever press (FR1) until 100 reinforcers were obtained. In the second phase, the session started with all of the lights OFF, and the two levers were retracted for a period of 3 min. Next, a non-contingent pellet was given at the same time than the house-light and the magazine-light was ON. When the rat nose-poked inside the food magazine, the magazine-light switched to OFF, and one of the two levers was then accessible. If the rat pressed the lever within a limited-hold of 30 s, the lever was retracted and 1 pellet was given. If the rat failed to respond (“omission”), the lever was also retracted but no pellet was given, and the house-light was switched OFF. Then, the next trial started with the illumination of the house-light and the magazine-light. The session ended when the animal performed 100 trials, and the session was repeated the next day until at least 60 reinforcers were obtained, and both levers were pressed at least 30 times. In the third phase of pre-training we introduced a limited-time of 10 s between the illumination of the house-light and the magazine-light and the nose-poke, the limited-hold period to press the lever was decreased from 30 s to 10 s, and the session finished after 60 trials or 90 min. There was no delay with any of the 2 levers in this pre-training.

After pre-training, the proper delay-discounting started using a between-sessions procedure. One lever (A, the “immediate”) produced a single pellet as a reinforcement after 1 lever press, and the other lever (B, the “delayed”) produced 4 pellets. The position of levers A and B (right/left) was counterbalanced across rats. During the days of testing, the delay of reinforcement presentation after the lever press was changed. For habituation to the training conditions, the delay for the corresponding lever was 0 s during 10 days, and the last session was chosen as a reference. On the next session the delay was increased to 10 s and maintained during 3 days more. Then the delay was increased to 20 s and maintained during 2 days more and later was increased to 40 s during 2 days more. The reference for the sessions with 10, 20 and 40 s of delay was the first session in those conditions. Finally, during 3 days the delay was decreased again to 0 s to ensure that the training and/or the passage of time did not modify the preference for the B lever when there was no delay (4 pellets). Lever A never had a delay. Each session consisted of 60 trials or 90 min in total. The basic procedure was the same as in the pre-training, except now half of the trials were “forced” and only 1 lever was accessible (15 times lever A and 15 times lever B), and the other half of the trials were “free” (“choice” or “decision” trials), where both levers were presented and the animal had to choose which to press. Each session was divided into five identical blocks of 12 trials in which the first six trials were of forced choice, and the last six were free choice trials. The start of each trial was signaled by turning on the house-light and the magazine-light. A nose-poke response was required in the food magazine for lever presentation. A failure to make a nose-poke in the food magazine or subsequently to depress the lever within 10 s (“omission”) terminated the trial. Thus, in each of the “free” trials, the subject had three possibilities: press lever A, press lever B or do not press the levers (omissions). When one of the two levers was pressed, the house-light was turned off and both levers were retracted. The food reward was then delivered, accompanied by the onset of the magazine-light, either immediately or after a delay (that was no signaled). When the food was collected, the magazine-light was switched off, and the chamber returned to the ITI state.

The main measures were: number of responses to the “delayed” lever in the “decision” trials; percent of responses to the “delayed” lever in the “decision” trials (excluding omissions); omissions; latency to collect the pellet in the “decision” trials; latency of response in the “decision” trials. Other measures were the duration of the session and the number of trials. The behavior during the pre-training and during the “forced” trials was also analyzed. All the testing lasted as a mean 42 days (including weekends with no testing).

*Gambling task*

The gambling task used assessed decision-making in a setting where the subject has to choose between different options that differ in the amount of reward and penalties (TO) associated^12^. In this cohort, 28 stress-naive, 27 CST and 30 UST animals were used. Due to the high number of subjects, the experiment was performed in two separate sub-sets of animals. One animal from the stress-naive group and another one from the UST group were removed from the experiment because they were not able to learn the task, giving a total of 83 subjects. In the statistical analysis 3 subjects were removed due to very extreme values (1 stress-naive and 2 CST), giving a total of 80 subjects.

We used a modification of a previous procedure^12^. During the first stage of pre-training (1-10 sessions, until they obtained at least 20 pellets and explored all holes) the session started with the 4 holes illuminated at the same time and the rat had to select one hole to nose-poke. The duration of the sessions in this stage was 30 min (or 20 pellets if before 30 min). The four holes (A, B, C, D) provided the same reinforcer (1 pellet). After the nose-poke, the selected hole remained illuminated until the pellet was collected. After the intake of the pellet, the 4 holes were again all illuminated. In the second stage of the pre-training (1-13 sessions) 2 consecutive nose-pokes in the same holes were required to get a reinforcer. In the last stage of pre-training (1-5 sessions) the session was divided into 2 parts, both of 15 min. In the first part, the reward was 2 pellets for all the holes (with a maximum of 40 pellets), and in the second part it was only 1 pellet (with a maximum of 20 pellets). The purpose of this stage was to habituate the animals to the rule that the amount of pellets could be variable.

One week later, the animals were run in the proper gambling test. The duration of the session was 1 h (or until obtaining 250 pellets). The response needed to get a reinforcer was always 2 consecutive nose-pokes in the same hole. In this session the contingencies for each hole were different. The hole A provided 2 pellets (100% trials) and at the same time a penalty of 222 s in 50% of the trials was programmed. The hole B provided 2 pellets (100% trials) and at the same time a penalty of 444 s in 25% of the trials was scheduled. The hole C provided 1 pellet (100% trials) and at the same time a penalty of 12 s in 25% of the trials was programmed. The hole D provided 1 pellet (100% trials) and at the same time a penalty of 6 s in 50% of the trials. The “penalty” was a TO period with no reinforcers available. During the TO the hole-light where the response was made remained illuminated at the same time that the other ones were OFF, and after the duration of the penalty the light remained OFF for 1 s. The penalty was assigned to each selection in a pseudo-random manner, because at the beginning of the session only reinforcers were available (and not penalties). After the 1 s with the light OFF, the 4 holes were again illuminated. During the TO the responses had no programmed consequences but were recorded. During the TO perseverative responses were also measured as the number of responses made in one hole after the reward was received and collected. One week after the gambling test, a retest was done with identical characteristics.

The main measures were: number of times that the animals performed two consecutive nose-pokes in a hole to get a reward (“valid” responses); number of nose-pokes per hole and total nose-pokes; percent of responses in each hole; number of reinforcers by hole and total reinforcers; percent of reinforcers by hole; number of perseveratives responses; number of responses during the TO; latency of response, and latency to collect the pellet. Other measures were: mean duration of a session and number of trials completed. All the testing lasted as a mean 23 days (including weekends with no testing).

*Probabilistic reversal learning*

The PRL task was used to evaluate behavioral flexibility in a probabilistic setting what makes the procedure more similar to human testing^13^. In this cohort, 8 stress-naive, 10 CST and 10 UST animals were used. A modification of a previous procedure^14^ was used with the main difference that the final probabilities associated with the “correct” and “incorrect” options were 95% and 25% respectively. The reward were 45 mg pellets (Ref F0021, Bioserv). The first day animals were introduced inside the operant boxes during 30 min with no levers available, 25 pellets inside the food magazine and the house-light on. If the animals did not eat all the available pellets they were re-introduced the next day in the box. Rats needed between 1-4 sessions to reach the criteria.

After that, in a first stage, rats were trained to press one of the two levers to receive one single pellet (FR1). Only one lever appeared at a time and the order of appearance was randomized across the session. Each lever was signaled by a light above that lever. A nose poke inside the food magazine finalized a trial and the next lever appeared. The duration of the session was 3 h and the animals had to receive 100 pellets to pass to the next stage. If the criterion was not reached, rats had 3-4 more sessions until they obtained 150 pellets in total in two consecutive sessions.

In a second stage, animals received a pellet after pressing one of the two levers in 50% of the cases (FR0.5). Only one lever appeared at a time and the order of appearance was randomized across the session. Each lever was signaled by the house-light. The lever was available only during 10 s and if the rat did not press it an omission was counted and a TO of 10 s started during which no lever was available and the house-light was off. The ITI was of 40 s. Each session lasted 90 trials. The rat passed to the next stage if 10 or less omissions were performed.

After pre-training, the proper PRL task started. In this stage the two levers were available at the same time. The “correct” lever was rewarded in 95% of the cases and the “incorrect” lever in 25% of the cases. At the start of the session one lever was randomly selected to become the “correct” lever and the other one to be the “incorrect” one (right/left counterbalanced). Once the rat pressed the “correct” lever in 8 consecutive trials (regardless of whether the choice was rewarded), the contingencies were reversed (“reversal”) and the “correct” lever became the “incorrect” and viceversa. If a rat performed an “incorrect” response (regardless of whether a reward was received) or an omission, the rat had to start a new series of 8 “correct” consecutive trials. The TO after an omission lasted 10 s and the ITI lasted 15 s. The duration of each session was of 200 trials or 50 min (whatever was first). The experiment finished when the rat performed 4 or more reversals in two consecutive sessions.

The main measures of cognitive flexibility were the number of reversals performed as a mean in a session and the number of perseverative errors in the last reversal. Perseverative errors were scored as the number of consecutive incorrect choices after the reversal and when the subject made a correct response perseverative errors were no longer scored. Additional measures were: number of rewarded and non-rewarded responses, number of responses to the “correct” and the “incorrect” lever, number of omissions, number of sessions to criterion, duration of the session, number of trials and latency of response. All the testing lasted as a mean 25 days (including weekends with no testing).

*Brain processing and in situ hybridization analysis*

Two different sub-sets of animals were perfused 3 days (short-term) or 33 days (long-term) after the end of the stress protocol. In each sub-set of this cohort 10 stress-naive, 10 CST and 10 UST animals were used. Animals were deeply anesthetized by isoﬂurane inhalation (Esteve Laboratories) and transcardially perfused, introducing ﬁrst a physiological saline solution (0.9% NaCl, 4ºC) for 2 min and then a ﬁxative solution (4% PFA + 3.8% Borax) for 12 min. Brains were then removed and post-fixed in the same solution and stored at 4ºC for 24 h. Then PFA was replaced by a cryoprotectant solution containing 30% sucrose in potassium phosphate-buffered saline (140 mM NaCl, 25mM potassium phosphate, pH 7.6) and brains were maintained in this condition for 48 h at 4°C. Brains were then frozen in dry ice cooled isopentane and preserved at −80°C until sections were obtained with a freezing cryostat. Then, 14 μm coronal sections were collected in antifreeze solution (30% ethylene glycol, 20% glycerol in 25 mM sodium phosphate buffer, pH 7.3) and stored at -20ºC until analysis. Sections from the striatum were collected.

D2R probe was generated by in vitro transcription from a plasmid containing rat full-length D2 dopamine receptor cDNA (Dr. David Grandy, Oregon Health Sciences University, USA). Probe sequence used is publicly available^15^. Antisense cRNA copies were labeled with Digoxigenin (DIG RNA Labeling Mix, Roche) using a transcription kit (SP6/T7 Transcription Kit, Roche). The transcription process was stopped by adding 40 μL of a sodium chloride–Tris–EDTA buffer solution (STE: 0.1 M NaCl, 10 mM Tris–HCl pH 8.0, 1 mM EDTA). Then, the product was heated during 5 min at 65°C. The probe was isolated through a gel ﬁltration column (mini Quick Spin RNAColumns, Roche) and stored at −20°C.

The protocol used for the chromogenic in situ hybridization (ISH) was adapted from a previous procedure^16^. Sections of regions of interest were mounted on positive-charged slides (Superfrost Plus, Thermo Scientiﬁc). All the solutions used before the hybridization with the D2R anti-sense probe were pretreated with diethylpyrocarbonate (DEPC) and sterilized. Tissue was first post-ﬁxed in 4% PFA + 3.8% Borax, rinsed in potassium phosphate-buffered saline and digested with 0.01 mg/mL of proteinase K (Roche, Penzberg, Germany) in an appropriate buffer (0.1 M Tris–HCl pH 8.0, 50 mM EDTA pH 8.0) for 15 min. After digestion, sections were rinsed in DEPC-treated water and acetylated during 10 min in 0.25% acetic anhydride in 0.1 M Triethanolamine pH 8.0. Finally, sections were washed in 2×saline-sodium citrate (SSC) solution (0.3M NaCl, 30mM sodium citrate), dehydrated through a graded concentration of ethanol and air-dried.

Thereafter, 150 μL of hybridization buffer (50% formamide,0.3 M NaCl, 10 mM Tris–Cl pH 8.0, 1 mM EDTA pH 8.0, 1×Denhardts, 10% dextranesulphate, yeast tRNA 1mg/ml, and 10 mM DTT) were added onto each slide and sealed with a coverslip. The dilution of the probe was 1:2000. Sections were incubated 20 h in a humid chamber at 60°C. After this time, sections were washed in 4×SSC and RNA digested with 0.02 mg/mL of RNase A (Roche) in an appropriate buffer (0.5 M NaCl, 10 mM Tris–HCl pH 8.0, 1 mM EDTA pH 8.0). After RNA digestion, sections were washed in descending concentrations of SSC (from 2× to 0.1×) and heated at 60°C in 0.1× SSC during 30 min. After the last wash in 0.1× SSC at 60°C sections were equilibrated in Tris–buffered saline with Tween20 (T-TBS; 0.1 M Tris–HCl pH 7.5, 0.15 M NaCl, 0.05% Tween20).

Sections were next incubated for 1 h in blocking buffer (2% bovine serum albumin [BSA] in T-TBS) before the immunodetection of DIG. Then, blocking buffer was removed and an alkaline phosphatase-conjugated anti-DIG antibody (anti-DIG-AP, Roche) was added at 1:500 in 1% fetal calf serum, 0.1% acetylated BSA, and 0.1% Tween20 in TBS using incubation chambers (CoverWell, Grace Bio-Labs). After incubation during 18 h at 4ºC, incubation chambers were removed and slides were washed in T-TBS.

A signal ampliﬁcation kit (NBT/BCIP tablets, Roche) was used to amplify the AP signal. Slices were first equilibrated in detection buffer (0.1 Tris-HCl, 0.1 NaCl, pH 9.5) and then incubated during 22-24 h at RT with the amplification kit. The reaction was stopped incubating the slices in an appropriate buffer (10mM Tris HCl, 1mM EDTA, pH 8.1). Finally the slices were rinsed with deionized water. After being air-dried, slices were covered with an aqueous mounting medium (Fluoromount, Sigma) and their edges sealed with rapid mounting medium (Entellan, Merck). Slides were stored at 4°C until image capture. All samples to be statistically compared were processed in the same assay to avoid interassay variability.

Following the reference of a stereotaxic atlas^17^, the same coordinates were used for each area in all histological analysis. The areas analyzed were (see Fig. 5): Dorsomedial (DMS), Dorsolateral (DLS), Ventromedial (VMS) and Ventrolateral (VLS) striatum, between Bregma + 1.44 and 2.28 mm. For the processing and the analysis of images, ImageJ public domain image processing software (FIJI v1.47f) was used. Images were previously coded before counting and analyzed at blind. Images were taken with 40x magnification using a bright-ﬁeld microscope (NIKON, Eclipse E400) coupled to a digital camera (NIKON, DMX 1200). Background signal was determined by the mean integrated density (ID) value of 15 unlabeled regions of interest (ROIs) per picture. ISH particles were considered positive cells when ID value of ROIs exceeded 3 standard deviations over the averaged background. The average of at least 12 ﬁelds (3 ﬁelds per hemisphere of 2 slices) per brain area and animal was used for the statistical analysis.

**Results**

*Shuttle-box behavior*

The number of escapes decreased along the sessions [SESSION: F(7,385)=52.8, p<0.001], following a linear [F(1,55)=99.9, p<0.001] and a quadratic trend [F(1,55)=68.6, p<0.001]. The number of null responses was very low (data not shown). As an additional index of learning, response latencies (time elapsed from the beginning of the tone to the behavior of crossing to the other compartment) across the 8 sessions were also measured (Suppl Fig. 1A). When a null response was performed in a given trial a latency of 20 s (duration of the CS + US) was scored. The SESSION factor was statistically significant for response latencies [F(7,385)=49.9, p<0.001] that decreased across sessions following a linear trend [F(1,55)=67.5, p<0.001] with a stabilization following a quadratic trend [F(1,55)=99.3, p<0.001].

For the trials where an avoidance response or an escape response was performed a separate analysis of avoidance and escape latencies were performed. Latency of avoidance responses slightly increased across sessions [SESSION: F(7,357)=3.98, p<0.001; linear trend: F(1,51)=10.6, p<0.01; in session 1= 4.7 + 0.2 s and in session 8: 5.7 + 0.2 s]. Latency of escape responses decreased across sessions [SESSION: F(7,385)=18.2, p<0.001; linear trend: F(1,55)=39.8, p<0.001; quadratic trend: F(1,55)=31.6, p<0.001; in session 1= 1.3 + 0.1 s and in session 8= 0.8 + 0.0 s].

The inter-crossings during the ITI in CST rats (Suppl Fig. 1B) also increased across sessions [SESSION: F(7,385)=14.5, p<0.001], following a linear [F(1,55)=38.3, p<0.001] and a quadratic trend [F(1,55)=29.1, p<0.001]. Obviously, the duration of the session [(Suppl. Fig. 1C; SESSION: F(7,385)= 31.7, p<0.001; linear trend: F(1,55)=67.6, p<0.001; quadratic trend: F(1,55)=58.0, p<0.001] and the total amount of shock received [Suppl. Fig. 1D; SESSION: F(7,385)=21.1, p<0.001; linear trend: F(1,55)=30.5, p<0.001; quadratic trend: F(1,55)=31.8, p<0.001] decreased across sessions.

**References**

1. Dalley, J. W., Cardinal, R. N. & Robbins, T. W. Prefrontal executive and cognitive functions in rodents: neural and neurochemical substrates. *Neurosci Biobehav Rev*, **28,** 771-784. (2004)

2. Lalanza, J. F. *et al.* Long-term moderate treadmill exercise promotes stress-coping strategies in male and female rats. *Sci Rep*. **5,** 16166 (2015).

3. LeDoux, J. E., Moscarello, J., Sears, R. & Campese, V. The birth, death and resurrection of avoidance: a reconceptualization of a troubled paradigm. *Mol Psychiatry*, **22,** 24-36 (2017).

4. Gorka, A. X, LaBar, K. S. & Hariri, A. R. Variability in emotional responsiveness and coping style during active avoidance as a window onto psychological vulnerability to stress. *Physiol Behav*. **158,** 90-99 (2016).

5. Blanchard, R. J. & Blanchard, D. C. Crouching as an index of fear. *J Comp Physiol Psychol*. **67**, 370-375 (1969).

6. Andero, R., Daviu, N., Escorihuela, R. M, Nadal. R. & Armario, A. 7,8-dihydroxyflavone, a TrkB receptor agonist, blocks long-term spatial memory impairment caused by immobilization stress in rats. *Hippocampus*. **22,** 399-408 (2010).

7. Muñoz-Abellán, C., Andero, R., Nadal, R. & Armario, A. Marked dissociation between hypothalamic-pituitary-adrenal activation and long-term behavioral effects in rats exposed to immobilization or cat odor. *Psychoneuroendocrinology.* **33,** 1139-1150 (2008).

8. Robbins, T. W. The 5-choice serial reaction time task: behavioural pharmacology and functional neurochemistry. *Psychopharmacology*. **163,** 362-380 (2002).

9. Fuentes, S. *et al.* Sex-dependent effects of an early life treatment in rats that increases maternal care: vulnerability or resilience? *Front Behav Neurosci*. **8,** 56 (2014).

10. Bari, A., Dalley, J. W. & Robbins, T. W. The application of the 5-choice serial reaction time task for the assessment of visual attentional processes and impulse control in rats. *Nat Protoc*. **3,** 759-767 (2008).

11. Dalley, J. W. & Robbins, T. W. Fractionating impulsivity: neuropsychiatric implications. *Nat Rev Neurosci.* **18,** 158-171 (2017).

12. Rivalan, M., Ahmed, S. H. & Dellu-Hagedorn, F. Risk-prone individuals prefer the wrong options on a rat version of the Iowa Gambling Task. *Biol Psychiatry*. **66,** 743-749 (2009).

13. Bari, A. *et al.* Serotonin modulates sensitivity to reward and negative feedback in a probabilistic reversal learning task in rats. *Neuropsychopharmacology*. **35**, 1290-1301 (2010).

14. Dalton, G. L., Wang, N. Y., Phillips, A. G. & Floresco, S. B. Multifaceted contributions by different regions of the orbitofrontal and medial prefrontal cortex to probabilistic reversal learning. *J Neurosci*. **36,** 1996-2006. (2016).

15. <https://www.ncbi.nlm.nih.gov/gene/24318>

16. Simmons, D. M, Arriza, J. L. & Swanson, L. W. A complete protocol for in situ hybridization of messenger RNAs in brain and other tissues with radio-labeled single-stranded RNA probes. *J Histotechnol*. **12,** 169-181 (1989).

17. Paxinos, G. & Watson, C. *The Rat Brain in Stereotaxic Coordinates*. (Academic Press, 2007).





Suppl. Figure 1. *Shuttle-box two-way active avoidance behavior in CST rats.*

CST rats (n=56) were exposed to a two-way active avoidance task in a shuttle-box for 8 sessions during the adolescent period. A. Latency (s) of response. B. Number of inter-crossings during the inter-trial interval (ITI). C. Duration (s) of the sessions. D. Total shock duration (s) received. The time effect is not represented. Means and SEM are represented.
